# Risk factors for sepsis and mortality in patients with emphysematous pyelonephritis : a series of 68 cases (case series)

**DOI:** 10.1097/MS9.0000000000001475

**Published:** 2023-11-07

**Authors:** Mohamed A. Bedoui, Ahmed Saadi, Selim Zaghbib, Seif Mokadem, Hamza Boussaffa, Amine Hermi, Haroun Ayed, Abderrazek Bouzouita, Marouene Chakroun, Riadh Ben Slama

**Affiliations:** Department of Urology, Faculty of Medicine of Tunis, Charles Nicolle Hospital, University of Tunis El Manar, Tunis, Tunisia

**Keywords:** case series, emphysematous pyelonephritis, mortality, risk factors, sepsis

## Abstract

**Background:**

Emphysematous pyelonephritis (EPN) is a rare and severe necrotizing infection of the kidney with a high rate of complications and mortality. Our aim was to investigate risk factors of urosepsis and mortality in case of EPN.

**Materials and methods:**

Between January 2010 and December 2022 the charts of patients diagnosed with EPN were retrospectively reviewed. Patients medical records were collected and data including demographics, BMI, EPN type, the organism causing the infection and biochemical variables were registered. The authors performed an univariate and multivariate logistic regression analysis for sepsis, septic shock, and mortality. Statistical significance was defined as a *P*-value of <0.05.

**Results:**

Our study included 68 patients (63% females, mean age 58.6 years old). Forty-eight patients (70.6%) had diabetes. Half of the patients (50%) presented with sepsis and 11 patients (16.2%) developed a septic shock. The following factors were associated with sepsis by univariate analysis: diabetes (*P*=0.01), higher blood sugar on admission (*P*=0.01), higher leukocytic count (*P*<0.001), higher lymphocytic count (*P*<0.001), and lower platelet to leukocytes ratio (*P*<0.001). Multivariate regression analysis revealed that the main risk factors of urosepsis were the leukocytic (OR: 85.7; 95% CI: 9.177–800.486; *P*<0.001) and lymphocytic count (OR: 6.65; 95% CI: 1.228–36.050; *P*=0.028). Neither of the variables was significantly associated with a higher risk of mortality.

**Conclusion:**

Leukocytic and lymphocytic count on admission are independent simple predictors for sepsis in patients with EPN.

## Introduction

Highlights

- Emphysematous pyelonephritis is a rare and severe necrotizing infection of the kidney.
- Half of the patients (50%) presented with sepsis and 11 patients (16.2%) developed a septic shock.
- Leukocytic count on admission and platelet to leukocyte count ratio seem to be significant risk factors for sepsis in patients with emphysematous pyelonephritis.

Emphysematous pyelonephritis (EPN) is a rare and severe necrotizing infection of the kidney characterized by the accumulation of gas within the collecting system, renal parenchyma, and/or perirenal tissues^1^. It is commonly seen in those with urinary tract obstruction with immune deficiency especially diabetic patients^2,3^. If not treated rapidly, the evolution is always fatal due to septic shock manifestation. In fact, up to the late 1970s, EPN was a life-threatening disease, with a mortality rate up to 78%^5^. Early detection and management of this condition is therefore of supreme importance in order to avoid its undesirable consequences especially septic complications. Recently, many series reported an improvement in mortality rate varying from 11 to 42%^3^. Risk factors of septic shock and mortality have been investigated by many authors including diabetes mellitus, disturbance of consciousness level, thrombocytopenia, hyperleukocytosis, platelet to leukocytes ratio (PLR), platelet to lymphocytes ratio (PLTR), hypoalbuminemia, and EPN radiological class^6–8^. Moreover, no recommendations are available for this serious disease.

In the present study, we have set ourselves the following two objectives:

1. To investigate the clinical characteristics and risk factors of urosepsis in patients with EPN.
2. To identify factors predictive of mortality in patients with EPN.

## Methods

Between January 2010 and December 2022, patients admitted with EPN were enrolled in this retrospective study. Clinical and demographic information, including age, sex, underlying medical conditions, laboratory findings, clinical features at initial presentation, imaging findings, type of management and patient outcomes were obtained from the medical charts. Clinical features included presentation delay, symptoms at presentation, hemodynamic status, and blood glucose levels on admission. The studied laboratory variables included leukocytic and lymphocytic count, platelet count, and serum creatinine levels. The platelet to leukocyte ratio was calculated for each patient. Results of bladder urine culture, pelvic urine culture in case of drainage and blood culture were assessed. A CT-scan was performed in all cases and patients were divided in four groups in accordance with the classification system of Huang and Tseng, which is based on the extent of air seen on CT: class 1, gas in the collecting system only; class 2, gas in the renal parenchyma without extension to the extrarenal space; class 3A, extension of gas or abscess to the perinephric space; class 3B, extension of gas or abscess to the pararenal space; and class 4, bilateral EPN or a solitary kidney with EPN^4^.

Thrombocytopenia was defined as a platelet count less than 120 000/ml. PLR was calculated by dividing the platelet rate by the leukocytic rate. PLTR was calculated by dividing the platelet rate by the lymphocyte rate. Sepsis was retained in patients with an increase of two points or more in the Sequential Organ Failure Assessment (SOFA) score. Septic shock was defined as a persistent hypotension requiring vasopressors or lactate greater than or equal to 2 mmol/l in septic patients. The management modalities consisted of antibiotics alone; percutaneous and/or endourological drainage with antibiotics and nephrectomy. A specific mortality rate was calculated based on patients in which mortality cause was related to septic shock complicating EPN.

In order to meet the first objective of our study, patients were divided into two groups according to whether or not they had developed urosepsis and to answer the second objective, we compared two groups of patients: those who had died as a result of EPN and those who had not. All factors were analyzed to determine which were significantly associated to urosepsis occurrence and specific mortality rate.

Continuous data were expressed as mean±SD or median and range when appropriate. Univariate analysis of predictors of sepsis was done using the *χ* ^2^ test, Student’s sample *t*-test or Anova test as appropriate. The receiver operating characteristics (ROC) curve was used to identify cut-off values for significant continuous variables in univariate analysis. Multivariate analysis was done using logistic regression analysis. All statistical tests were carried out using IBM ʻSPSSʼ statistics version 25, with a *P*-value <0.05 was considered significant.

This case series has been reported in line with the PROCESS Guideline^16^.

## Results

Sixty-eight patients were included in the study. The mean age of the patients was 58.6 years (22–88). There were 43 women and 25 men. The mean presentation delay was 5.96 days (1–30). A total of 48 patients (70.6%) had diabetes mellitus. Obstructive uropathy was found in 42 patients (61.8%) and was due to lithiasis in 36 cases (52.9%). In diabetic patients, mean blood glucose levels on admission was 14.84 mmol/l (7–33). The mean leukocyte count at admission was 20 296/μl (3760–52 580). The mean lymphocytic count was 3068/μl (1220–7520). Thrombocytopenia was present in eight out of 68 (11.7%). The mean platelet to leukocyte ratio was 20.68. Urine culture was positive in 47 patients (69.1%), and 11 patients had a polymicrobial infection (16.1%). Blood cultures were performed in 21 patients (30.8%) and was positive in four cases (5.9%). *Escherichia coli* was the most common organism cultured from specimens of urine and blood and was present in 29 (42%) patients. The other organisms included *Klebsiella Pneumoniae* (7.4%), *Enterococcus Faecalis* (5.9%), *Staphylococcus Aureus* (4.4%), and *Candida* species (2.9%). Nine patients (13.2%) had bilateral involvement. In the other patients, the left side was more frequently involved (51.5%) than the right side (35.3%). As per Huang and Tseng classification, 34 patients had class 1 (50%), 11 had class 2 (16.2%), 13 had class 3 (19.1%), and 10 had class 4 CT imaging findings (14.7%).

Management was either conservative or radical. Radical treatment was primary nephrectomy or secondary salvage nephrectomy in cases of failure of conservative treatment. One patient had a salvage nephrectomy without drainage (1.5%). The conservative attitude in responsive cases consisted in antibiotic therapy alone or/and upper urinary tract (UUT) drainage (endourological or percutaneous). Antibiotic therapy alone was used in 10 cases (14.7%) while endourological drainage of UUT was associated to medical treatment in 55 cases (80.9%). A percutaneous drainage was performed in three patients (4.4%). The combination of a third-generation cephalosporin and an aminoglycoside was the most common initial empiric antimicrobial therapy, accounting for 74%, and was subsequently adapted on the basis of culture results for oral relay, mainly using cotrimoxazole (55%), fluoroquinolones (32%), and oral third-generation cephalosporins (13%). An initial combination of imipenem and an aminoglycoside was used in 26% of patients known to carry multiresistant bacteria or in cases of septic shock.

On admission, 34 patients (50%) had sepsis criteria and 11 patients (16.2%) developed a septic shock. Six patients presented a septic shock refractory to conservative management requiring nephrectomy with a delay of 4.66 days.

**Figure 1:**
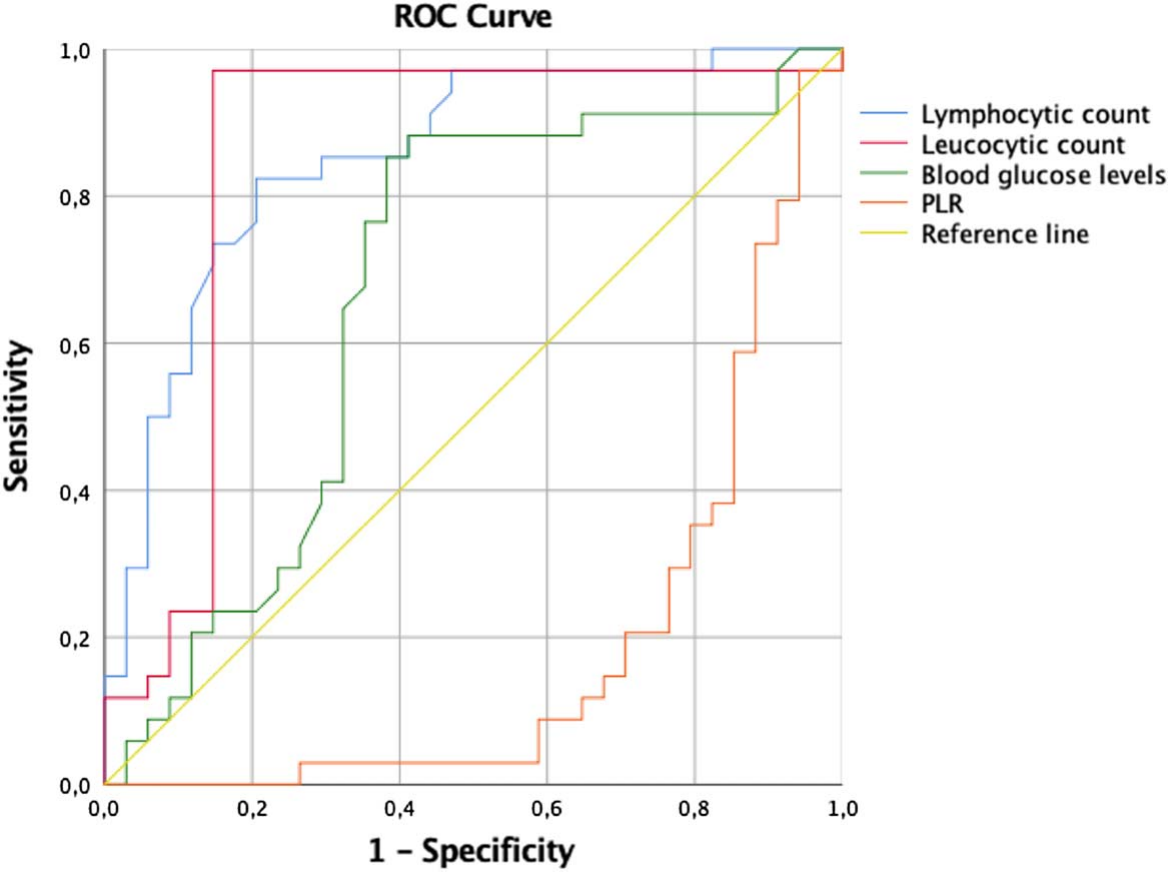
ROC curve of biological parameters (Blue=lymphocytic count, Red=Leukocytic count, Green=Blood glucose levels, Orange=PLR) for sepsis prediction.

In univariate analysis, diabetes Mellitus (*P*=0.01), higher blood glucose levels on admission (*P*=0.01), higher leukocytic count (*P*<0.001), higher lymphocytic count (*P*<0.001), lower PLR (*P*<0.001) and lower PLTR (*P*=0.008) were significantly associated with sepsis occurrence. There were no significant differences between the two groups with respect to age, urological history, causative organisms, thrombocytopenia, acute kidney injury, serum creatinine level, presence of hematuria, urinary tract obstruction, type of EPN and urolithiasis (Table 1). In multivariate logistic regression analysis, leukocytic (OR: 85.7; 95% CI: 9.177–800.486 ; *P*<0.001) and lymphocytic count (OR: 6.65 ; 95% CI: 1.228–36.050 ; *P*=0.028) were independent risk factors for sepsis. The AUC of the ROC curve was 0.853 for both of the two independent prognostic factors (Fig. 1).

**Table 1:** Univariate analysis of sepsis prediction according to patient’s clinical, biological, and radiological variables.

Variables	UroSepsis	No urosepsis	*P*
Age (years)	58.85	61.14	0.496
Presentation delay (days)	7.29	8.76	0.405
Sex			
Male	14 (41%)	11 (32%)	0.451
Female	20 (59%)	23 (68%)	
Diabetus mellitus			
Yes	28 (82%)	18 (53%)	0.010
No	6 (8%)	16 (47%)	
Urological records			
Yes	19 (56%)	23 (68%)	0.139
No	15 (44%)	11 (32%)	
Obstructive pyelonephritis			
Yes	20 (59%)	24 (71%)	0.310
No	14 (41%)	10 (29%)	
Hematuria			
Yes	1 (3%)	4 (12%)	0.163
No	33 (97%)	30 (88%)	
Blood sugar on admission (mmol/l)	15.83	11.52	0.010
Leukocytic count	21 058	11 955	<0.001
Lymphocytic count	3518	2086	<0.001
Platelet count	227.67 ×10^3^	274.00 ×10^3^	0.176
PLR	11.71	29.64	<0.001
PLTR	79.58	166.77	0.008
Urine culture results			
Positive	23 (68%)	24 (71%)	0.759
Negative	5 (15%)	7 (21%)	
Polymicrobial	6 (17%)	5 (8%)	
EPN class			
I	15 (44%)	19 (56%)	0.265
II	8 (24%)	4 (12%)	
IIIA	1 (3%)	0 (0%)	
IIIB	3 (9%)	7 (21%)	
IV	7 (10%)	4 (11%)	

Three patients died because of a refractory septic shock (4.4%). One patient after conservative management and two patients in whom UUT drainage failed died after salvage nephrectomy. The nonspecific mortality rate was 1.92%, in only one patient who passed out after acute pancreatitis stage 5. DM history, blood sugar on admission, leukocytic rate, lymphocytic rate, thrombocytopenia, PLR, creatinine serum level, PNE type and EBSL-secreting bacteria were not associated with a higher rate of mortality (*P*=0.372, *P*=0.511, *P*=0.555, *P*=0.533, *P*=0.865, *P*=0.220, *P*=0.699, *P*=0.884 and *P*=0.288, respectively).

## Discussion

EPN is a life-threatening disease mainly with its fatal septic complications. The present study sought to identify factors that were predictive of sepsis and mortality in case of EPN. Diabetes mellitus appeared to be the most common risk factor for EPN^9^. In fact, a high tissue glucose level might provide a favorable environment for the growth of gas-producing bacteria. In fact, almost 95% of patients with EPN suffer from underlying uncontrolled diabetes with high blood sugar levels^4^. DM was found to be associated with an increased risk of sepsis in the present study (*P*=0.02). Although, some recent series found that there was no association between mortality and diabetes mellitus in patients with EPN^3,6,10^. Unlike Elbaset *et al*.^8^ who found that male sex was significantly associated with sepsis (*P*=0.0001), there was no significant association between sex, age, and other comorbidities with sepsis in our series. Moreover, blood glucose levels on admission was significantly predictive of sepsis occurrence (*P*=0.03).

Leukocyte plays an important role in the systemic inflammatory response to infection. Most of the sepsis scoring systems use either leucocytosis (more than 12 000/l) or leukopenia (lower 4000/l) as a severity index^11^. In EPN, some authors established that a higher white blood cells count was significantly associated with severe infection^8^. Similarly, we found that hyperleukocytosis and higher lymphocyte blood cells count were predictors of urosepsis in univariate and multivariate analysis.

Platelets play an active role in antimicrobial host defense, inflammation, and tissue repair induction as well as their participation in hemostasis. By making cell to cell contacts with leukocytes and endothelial cells, platelets support leukocytes in arrest and transmigration. Platelets can bind and internalize pathogens and release microbicidal proteins that kill certain bacteria and fungi^12^. Therefore, platelets may become a defense weapon when irritated by bacteria. In the case of EPN, thrombocytopenia is usually caused by disseminated intravascular coagulation or by platelet consumption. Many series have shown that thrombocytopenia is considered as an important prognostic factor in EPN^3,8^. Whereas, in our series, a lower platelet count was not significantly associated with a higher risk of sepsis or mortality.

PLR is a new marker that copies a patient’s baseline health status. Patients with a lower ratio were more likely to have more comorbidity. In recent studies, lower PLR was associated with poor outcomes in many diseases like colon, lung, and testicular germ cell tumors^12^. Garbens *et al*.^13^ correlated the rate of UTI and sepsis postradical nephrectomy with the PLR and reported that a higher PLR was associated with a lower risk of 30-day postoperative infectious complications. To the best of our knowledge, only one study investigated the relationship between PLR and sepsis occurrence in patients with EPN and found that PLR was an independent simple predictor for sepsis and mortality in these patients^8^. Equivalently, in the current study, PLR was inversely associated with sepsis development.

Unlike other series that found that bacteria type could influence EPN severity, with especially Klebsiella infection^14^, the type of organism was not a risk factor of severity of mortality. Radiologically, Huang and Tseng described in 2000 four distinct radiological classifications of EPN based on the extent of gas seen on CT^4^. There was a tendency for higher mortality in patients with extensive disease, and patients with type 1 EPN had the best prognosis. As far as many recent studies which reported no association found between EPN classification and disease severity or mortality. In the present study, the outcomes of patients with classes 3 and 4 EPN were not statistically different from those with classes 1 and 2 disease. On the contrary, Torres-Mercado *et al*.^6^ reported that Huang’s radiological class 3 was statistically associated with adverse outcome (*P*<0.0001) while Falagas *et al*.^3^ concluded that EPN class I was significantly associated with higher mortality. A recent meta-analysis of 1303 patients, 177 of whom died after EPN, showed that patients with stage 3 or 4 according to the Huang-Tseng classification had twice the risk of death compared with other patients^15^.

In the current study, EPN management was either conservative or radical. The conservative attitude in responsive cases consisted in antibiotic therapy alone or/and UUT drainage (endourological or percutaneous). In refractory cases, nephrectomy was realised. The mortality rate was 3.82%. In a large meta-analysis discussing the risk of mortality in 175 patients with EPN, the incidence of mortality was 25%, ranging from 11 to 42%^3^.

To the best of our knowledge, this is the first study to assess the risk factors for sepsis development in EPN. Yet, it has many limitations like the limited number of patients, which is explained by the relative rarity of the disease. In addition to that, it is a retrospective study with the inherent nature of selection bias. Lack of available data regarding LDH and differential leukocytic count is one of the limitations, but this is because it was not a routine evaluation in our hospital.

## Conclusion

Leukocytic and lymphocytic counts at admission appear to be major risk factors for sepsis in patients with EPN. When treating patients with this devastating infection, these findings might be taken into account and can be used as warning indications to immediately drain the afflicted kidney.

## Ethical approval

All procedures performed in studies involving human participants were in accordance with the ethical standards of the institutional and/or national research committee and with the 1964 Helsinki Declaration and its later amendments or comparable ethical standards. The institutional ethical committee stated that there was no need to have an ethical approval.

## Consent

Written informed consent was obtained from the patient for publication and any accompanying images. A copy of the written consent is available for review by the Editor-in-Chief of this journal on request.

## Sources of funding

This research did not receive any specific grant from funding agencies in the public, commercial, or not-for-profit sectors.

## Author contribution

A.S., M.A.B., and S.M.: study conception and design; A.S., M.A.B., S.Z., S.M., H.B., and A.H.: acquisition of data; A.S., M.A.B., S.Z., and S.M.: analysis and interpretation of data; A.S. and M.A.B.: drafting of manuscript; A.S., H.A., A.B., M.C., and R.B.S.: critical revision of the manuscript; H.A., A.B., M.C., and R.B.S.: administrative support. All authors contributed in final approval of manuscript.

## Conflicts of interest disclosure

The authors have no conflicts of interest to declare.

## Research registration unique identifying number (UIN)

It is not a prospective study.

## Guarantor

Mohamed Ali Bedoui.

## Data availability statement

The data that support the findings of this study are available from the corresponding author upon reasonable request.

## Provenance and peer review

My paper was not invited.

## Authorship statement

All the authors meet the ICMJE authorship criteria.
